# Contribution of direct-drinking water to calcium and magnesium and the influence on the height in school-age children

**DOI:** 10.3389/fnut.2024.1434952

**Published:** 2024-10-16

**Authors:** Hongru Gu, Yijing Gong, Zhao Li, Yanqiu Zhang, Jin Wu, Yi Wang, Min Ni, Jun Zhang, Hai Jiang

**Affiliations:** Taicang Center for Disease Control and Prevention, Taicang, China

**Keywords:** direct-drinking water in school, children, calcium, magnesium, contribution rate, height

## Abstract

**Objective:**

To estimate the contribution of direct-drinking water provided in school to dietary intake (DI) and recommended nutrient intake (RNI) of calcium and magnesium, and to explore its influence on the height in school-age children.

**Methods:**

Tap water and direct-drinking water samples were collected from schools in Taicang City to test the contents of calcium and magnesium, and compared by one-way ANOVA and *post-hoc* test. Contribution rates of direct-drinking water to DI and RNI were calculated by using the contents of calcium and magnesium and data from the *Nutrition and Health Status Survey 2021*. A retrospective cohort was conducted among 4,850 first-grade children consuming direct-drinking water in Taicang City from 24 primary schools in 2019. Group 1 (1,070 boys and 946 girls) consumed UF-process water with normal calcium and magnesium contents and Group 2 (1,548 boys and 1,286 girls) consumed NF/RO-process water with very low calcium and magnesium contents. During 2019–2023, the height and height growth were analyzed with the Student’s *t*-test.

**Results:**

The highest calcium content was examined in tap water samples, followed by direct-drinking water samples supplied through a UF, NF, and RO system (*F* = 1,227.725, *p* < 0.001). The highest magnesium content was examined in water supplied through a UF system, followed by that through a tap, NF and RO system (*F* = 146.504, *p* < 0.001). Calcium and magnesium contents in direct-drinking water supplied through a UF system changed little compared with those in tap water, which were significantly reduced in direct-drinking water supplied through a NF and RO system. The contribution rates of direct-drinking water to DI of calcium and magnesium were 8.95 and 2.78%, respectively, and those to RNI of calcium and magnesium were 2.63 and 1.96%, respectively. There were no significant differences in the height and height growth of first-grade children drinking water supplied through the UF system vs. NF/RO system (*p* > 0.05).

**Conclusion:**

Direct-drinking water processed through a NF or RO system should be cautiously adopted in primary and secondary schools. A UF system is preferred in schools where no health concerns are associated with water quality.

## Introduction

Direct-drinking water is featured by non-pollution, enrichment of beneficial mineral elements similar to the human body and weak alkalinity, which can be consumed directly. With the rapid development of economy and technology and the increasing demand for high-quality drinking water, direct-drinking water has been gradually used in China (1). A series of laws and regulations have been released to standardize drinking water safety. In China, the *National Food Safety Standard for Drinking Water Quality (GB5749-2022)* and the *Water Quality Standards for Fine Drinking Water (CJ 94-2005)* specify the water quality standards for drinking purified water (2, 3).

At present, direct-drinking water systems have been introduced in schools. According to the differences in water filtration, commonly used direct-drinking water systems are classified into the ultrafiltration (UF) system, nanofiltration (NF) system and reverse osmosis (RO) system. They are all pressure-driving techniques, but differ from the transport mechanisms, solute rejections and fouling phenomena. The size of removed materials during the process of filtration is entirely dependent on the size of the filter pores. Specifically, the pore size of a UF, NF, and RO filters is about 0.01, 0.001, and 0.0001 μm, respectively (4). A UF system removes larger particles, but dissolved substances remained (5). Most of organic molecules and natural organic matter can be removed through a NF system, softening hard water (6). A RO system is capable of removing all viruses and organic molecules, and most of minerals (7). Considering the broad use of direct-drinking water systems in schools, water safety have slipped into the spotlight of research. A survey in elementary and middle schools in Shanghai suggested that the direct-drinking water systems should be kept far away from toilets and at room temperature or heated over 60°C (8). Chen et al. (1) analyzed colony forming units (CFU) in schools from Nanjing City, and reported that CFU increases when the filter has not been replaced for 3 months. The safety of direct-drinking water, especially in school, should be highly concerned.

Calcium and magnesium are two essential nutrients responsible for bone health (9). They are multifunctional to support bone strength, sleep quality, immune system and blood sugar (10). Magnesium deficiency is believed as a risk factor for osteoporosis (11). School-age children are in a period of rapid physical development, and calcium and magnesium supplementations greatly increase the height growth (12, 13). However, research on the impact of direct-drinking water on children’s health is scant. Chinese children spend two-thirds of their non-sleep time at school, and therefore, direct-drinking water at school accounts for a large part of their daily water consumption (14). Children gain height rapidly during their teenage years (15), and differences in height caused by mineral deficiencies during this period would be easier to detect. An eco-epidemiological study in Chongqing City demonstrated that drinking RO-process direct-drinking water may negatively influence the height growth and increase the incidence of dental caries in school-age children (14). Government departments have shown concerns on this issue. In 2013, the Shanghai Municipal Education Commission and other departments stipulated that RO-process direct-drinking water systems are prohibited in schools (16). In 2019, the National Ministry of Education recommended the use of RO-process direct-drinking water systems only in areas with large raw water pollution, and nanofiltration or ultrafiltration systems are replaced after qualifying the raw water (17). Since 2015, direct-drinking water systems have been deployed in schools from Taicang City on a large scale. It is necessary to illustrate the potential influence of direct-drinking water on the health of school-age children.

This study aims to examine the calcium and magnesium contents of tap water and direct-drinking water of schools in Taicang City, and to estimate the contribution of direct-drinking water provided in school to dietary intake (DI) and recommended nutrient intake (RNI) of calcium and magnesium. Secondary outcome was the influence of calcium and magnesium contents in direct-drinking water on the height of school-age children. We explored the under-researched area and our findings are expected to provide references for selecting optimal direct-drinking water systems in local schools.

## Methods

### Study design

**Figure 1:**
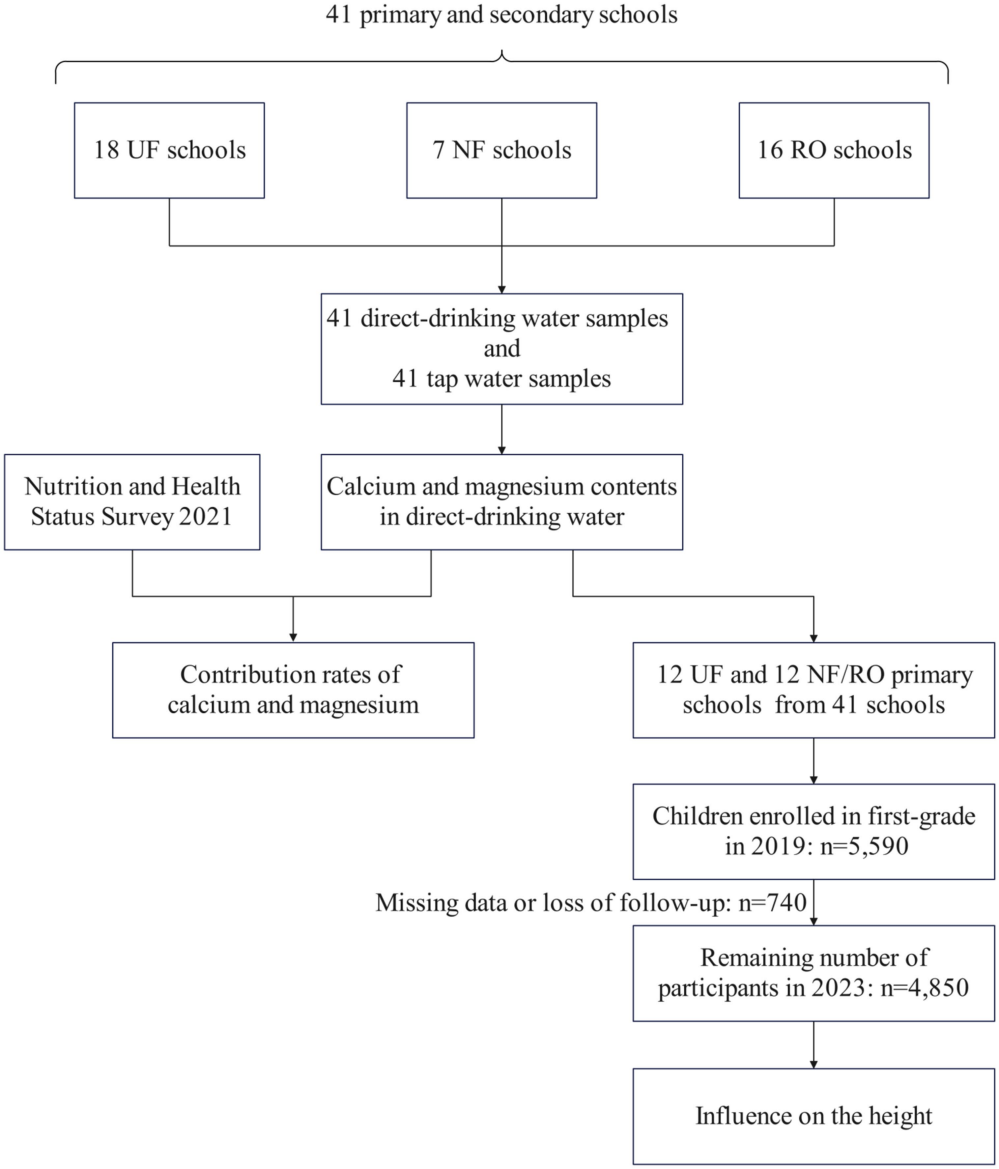
A flow chart of study design.

Measurement of water samples and calculations of DI and RNI was conducted through an observational cross-sectional study. The influence on the height in school-age children was analyzed via a retrospective cohort study. Tap water and direct-drinking water samples from 41 schools were collected to examine the calcium and magnesium contents. The contribution rates of direct-drinking water provided in school to dietary intake (DI) and recommended nutrient intake (RNI) of calcium and magnesium were estimated. In addition, a total of 4,850 children from 24 primary schools were divided into normal water calcium and magnesium intake group and very low water calcium and magnesium intake group. Data about their height were continuously tracked for 5 years to explore the influence of calcium and magnesium contents in direct-drinking water on children’s height. The flow chart of study design was illustrated in Figure 1.

### Collection of water samples

From February to March 2023, a direct-drinking water machine after changing the filter membrane for 1 week and a tap with a large water consumption were randomly selected. One water sample from the direct-drinking water machine and one tap water sample were collected from each school, for a total of 41 primary and secondary schools. Among the 41 water samples from the direct-drinking water machine, there were 18, 7, and 16 samples collected from water supplied through a UF, NF, and RO system, respectively. Collection, preservation, and transportation of water samples were strictly performed following the *Standard examination methods for drinking water (GB/T 5750-2023)* (18).

### Measurements

Calcium and magnesium contents in water samples were measured using a flame atomic absorption spectrophotometer (FAAS, ZA3000 series, Hitachi, Japan) based on the *National food safety standard—Methods for examination of natural mineral drinking water (GB 8538-2022)* (19). Detection limits of calcium and magnesium were 0.05 mg/L and 0.02 mg/L, respectively. The potassium, sodium, calcium, and magnesium mixed sample for water testing (GSB 07-3185-2014; Batch No., 202622) was adopted for quality control.

### Calculations of DI and RNI

Contribution rates of direct-drinking water to dietary intake (DI) and recommended nutrient intake (RNI) of calcium and magnesium were calculated using the Formulas 1, 2, respectively.


(1) $$C_{DI}=\frac{C_{ddw}\times V}{1000\times DI}\times 100\%$$

(2) $$C_{RNI}=\frac{C_{ddw}\times V}{1000\times RNI}\times 100\%$$

In Formula 1, *C_DI_* (%) denotes the contribution rate of direct-drinking water to dietary intake of calcium/magnesium; *C_ddw_* (mg/L) denotes the calcium/magnesium content in water samples; *V* (mL/day) and *DI* (mg/day) are water intake volume at school and dietary intake of calcium/magnesium recorded in the *Monitoring Site in Taicang City, Jiangsu Resident Nutrition and Health Status Survey 2021* (20), respectively. In Formula 2, *C_RNI_* (%) denotes the contribution rate of direct-drinking water to the recommended intake of calcium/magnesium (RNI) described in the *Dietary Guidelines for Chinese Residents (2022)* (21) (Table 1).

**Table 1:** Water intake volume at school (*V*), dietary intake of calcium/magnesium (*DI*), and recommended intake of calcium/magnesium (*RNI*) in children in primary and secondary schools in Taicang City.

	V (mL/day)	Calcium		Magnesium	
		DI (mg/day)	RNI (mg/day)	DI (mg/day)	RNI (mg/day)
Age (years)					
4–6	542.68	271.33	800	124.16	160
7–10	592.06	262.26	1,000	141.44	220
11–13	644.20	285.83	1,200	152.95	300
14–16	649.59	356.43	1,000	189.85	320
Sex					
Male	649.34	292.90	1,000	158.28	220
Female	574.45	295.15	1,000	152.02	220
Total	618.35	294.07	1,000	155.02	220

RNI stratified by the sex and the total cohort is defined as the standards in the 7–10 years of age group.

### A retrospective cohort of first-grade children for tracing the height and height growth

Physical examinations of first-grade children were performed by pre-trained healthcare providers in the community health centers from September to November annually during 2019–2023. An inquiry was conducted during the physical examination. Those transferred to another school, absented from annual physical examination, combined with development-associated diseases (e.g., malnutrition, chronic digestive disorders, and metabolic syndrome), or developed a family history of these diseases were excluded. During the investigation period for tracing their height and height growth, direct-drinking water processed through the filtration system in the school remained unchangeable. According to Chongqing (14) and Gambian (22) studies, and the mean and standard deviation of children’s height in Taicang City, we assumed a height growth difference of 0.5 cm. Assuming *α* = 0.05 and *β* = 0.2, GPower3.1 was used to calculate the sample size of 793 in each group. Through the school physical examination system, we obtained the height data of all first-grade children in primary schools in Taicang City with a consumption of direct-drinking water. The initial sample consisted of 5,590 children. Due to missing data of physical examination or follow-up, 740 children were excluded. Finally, 2,618 boys (Group 1 = 1,070, Group 2 = 1,548) and 2,232 girls (Group 1 = 946, Group 2 = 1,286) remained in the cohort.

### Statistical processing

Statistical analysis was performed using Statistical Product and Service Solutions (SPSS) 26.0. Normally distributed data were expressed as mean ± standard deviation ($\bar{x}$±SD). Normality was tested by graphical methods (histograms, boxplots, and Q-Q plots) and checked using skewness and kurtosis (23). Differences in calcium and magnesium contents were compared by one-way ANOVA and *post-hoc* test. Differences in urban/rural were compared by Chi-square test. Differences in age, weight, height, and height growth were compared by Student’s *t*-test. A significant difference was determined at *p* < 0.05.

## Results

### Calcium and magnesium contents in direct-drinking water

The highest calcium content was examined in tap water samples, followed by direct-drinking water samples supplied through a UF, NF and RO system (*F* = 1,227.725, *p* < 0.001). The highest magnesium content was examined in water supplied through a UF system, followed by that through a tap, NF and RO system (*F* = 146.504, *p* < 0.001). Multiple comparisons showed that there was no significant difference in the magnesium content between tap water samples and those supplied through a UF system (*p* > 0.05). In addition, no significant differences were detected in calcium and magnesium contents between water samples supplied through the NF vs. RO system (*p* > 0.05, Table 2).

**Table 2:** Calcium and magnesium contents in direct-drinking water processed by different filtration systems.

	Calcium (mg/L)	Magnesium (mg/L)
Tap water	45.85 ± 4.04^a^	6.61 ± 2.25^a^
UF-process water	42.55 ± 3.39^b^	6.98 ± 2.63^a^
NF-process water	3.29 ± 2.93^c^	0.17 ± 0.28^b^
RO-process water	2.66 ± 1.99^c^	0.08 ± 0.11^b^
*F* value	1,227.725	146.504
*p* value	<0.001	<0.001

Labeling the same letter a, b, or c in the same column indicates there is no significant difference between two groups (*p* > 0.05).

### Contribution rates of calcium in direct-drinking water to DI and RNI

The contribution rate of calcium in direct-drinking water supplied through a UF system provided in primary and secondary schools in Taicang City to DI ranged from 7.75 to 9.61%. Stratified by age, the lowest and highest contribution rates were detected in the age groups of 14–16 years and 7–10 years, respectively. In addition, the contribution rate of calcium in direct-drinking water supplied through a UF system to RNI was 2.28–2.89%, achieving the bottom and peak in the age groups of 11–13 years and 4–6 years, respectively.

Moreover, contribution rates of calcium in direct-drinking water supplied through a UF system to both DI (9.43% vs. 8.28%) and RNI (2.76% vs. 2.44%) were higher in male school-age children than those of female children. Overall, the contribution rates of calcium in direct-drinking water supplied through the NF and RO system to DI were 0.69 and 0.56%, respectively; and those to RNI were 0.20 and 0.16%, respectively (Table 3).

**Table 3:** Contribution rate of calcium in direct-drinking water in school-age children from Taicang City.

	*C_DI_* (%)			*C_RNI_* (%)		
	UF	NF	RO	UF	NF	RO
Age (years)						
4–6	8.51	0.66	0.53	2.89	0.22	0.18
7–10	9.61	0.74	0.60	2.52	0.19	0.16
11–13	9.59	0.74	0.60	2.28	0.18	0.14
14–16	7.75	0.60	0.48	2.76	0.21	0.17
Sex						
Male	9.43	0.73	0.59	2.76	0.21	0.17
Female	8.28	0.64	0.52	2.44	0.19	0.15
Total	8.95	0.69	0.56	2.63	0.20	0.16

C_DI_, Contribution rate of calcium to dietary intake; C_RNI_, Contribution rate of calcium to recommended nutrition intake; UF, Ultrafiltration; NF, Nanofiltration; RO, Reverse osmosis.

### Contribution rates of magnesium in direct-drinking water to DI and RNI

Magnesium in UF-process direct-drinking water provided a 2.39–3.05% of contribution rate to DI, and 1.42–2.37% of contribution rate to RNI in primary and secondary school children in Taicang City. The lowest and highest contribution rates to either DI or RNI were detected in the age groups of 14–16 years and 4–6 years, respectively.

Magnesium in UF-process direct-drinking water provided a higher contribution rate to DI (2.86% vs. 2.64%) and RNI (2.06% vs. 1.82%) in male school-age children than females. Overall, the contribution rates of magnesium in direct-drinking water supplied through the NF and RO system to DI were 0.07 and 0.03%, respectively; and those to RNI were 0.05 and 0.02%, respectively (Table 4).

**Table 4:** Contribution rate of magnesium in direct-drinking water in school-age children from Taicang City.

	*C_DI_* (%)			*C_RNI_* (%)		
	UF	NF	RO	UF	NF	RO
Age (years)						
4–6	3.05	0.07	0.03	2.37	0.06	0.03
7–10	2.92	0.07	0.03	1.88	0.05	0.02
11–13	2.94	0.07	0.03	1.50	0.04	0.02
14–16	2.39	0.06	0.03	1.42	0.03	0.02
Sex						
Male	2.86	0.07	0.03	2.06	0.05	0.02
Female	2.64	0.06	0.03	1.82	0.04	0.02
Total	2.78	0.07	0.03	1.96	0.05	0.02

C_DI_, Contribution rate of magnesium to dietary intake; C_RNI_, Contribution rate of magnesium to recommended nutrition intake; UF, Ultrafiltration; NF, Nanofiltration; RO, Reverse osmosis.

### Influence of direct-drinking water provided in school on the height and height growth

According to the exposure to direct-drinking water with very-low calcium and magnesium contents or not, a cohort of 4,850 first-grade children receiving physical examinations in Taicang City were divided into UF group and NF/RO group. There were 2,016 children in UF group, including 1,070 boys and 946 girls. A total of 1,548 boys and 1,286 girls were enrolled in NF/RO group. There were no significant differences in the region, age, or weight between the two groups in baseline (*p* > 0.05, Table 5). For consecutive 5 years from 2019 to 2023, there was no significant difference in the height of boys between UF group and NF/RO group (*p* > 0.05). Except for a significant difference in the height of girls between groups in 2019 (*t* = −3.001, *p =* 0.003), no significant difference was detected during 2020–2023 (*p* > 0.05). The mean height growth in the cohort during 2019–2023 was 0.2 cm higher in UF group than that of NF/RO group, although a significant difference was not detectable (*p* > 0.05, Table 6).

**Table 5:** Baseline characteristics of children from the study by different type of direct-drinking water in 2019 [mean ± SD or *n* (%)].

	Male (*n* = 2,618)			Female (*n* = 2,232)		
	UF (*n* = 1,070)	NF/RO (*n* = 1,548)	*p*	UF (*n* = 946)	NF/RO (*n* = 1,286)	*p*
Region						
Urban	442 (41.3)	672 (43.4)	0.285	426 (45.0)	568 (44.2)	0.685
Rural	628 (58.7)	876 (56.6)	/	520 (55.0)	718 (55.8)	/
Age (years)	6.7 ± 0.3	6.7 ± 0.3	0.703	6.6 ± 0.3	6.7 ± 0.3	0.312
Weight (kg)	24.1 ± 4.9	24.2 ± 5.7	0.583	22.6 ± 4.4	22.9 ± 4.6	0.068

UF, Ultrafiltration; NF, Nanofiltration; RO, Reverse osmosis.

**Table 6:** The height and height growth in first-grade children in Taicang City influenced by direct-drinking water in school from 2019 to 2023 (*n* = 4,850).

	Male (*n* = 2,618)				Female (*n* = 2,232)			
	UF (*n* = 1,070)	NF/RO (*n* = 1,548)	*t*	*p*	UF (*n* = 946)	NF/RO (*n* = 1,286)	*t*	*p*
Height (cm)								
2019	121.7 ± 5.2	122.0 ± 5.5	−1.373	0.170	120.4 ± 5.5	121.1 ± 5.3	−3.001	0.003
2020	128.0 ± 5.6	127.6 ± 5.7	1.909	0.056	126.5 ± 5.9	126.6 ± 5.5	−0.220	0.826
2021	133.7 ± 6.1	133.6 ± 6.1	0.562	0.574	132.4 ± 6.4	132.7 ± 6.0	−1.028	0.304
2022	139.4 ± 6.4	139.5 ± 6.5	−0.434	0.664	139.2 ± 7.3	139.7 ± 6.8	−1.721	0.085
2023	145.0 ± 7.0	145.1 ± 7.0	−0.262	0.793	146.1 ± 7.9	146.6 ± 7.1	−1.484	0.138
Height growth (cm)								
2019–2023	23.3 ± 3.4	23.1 ± 3.9	1.517	0.129	25.8 ± 4.4	25.6 ± 4.1	1.148	0.251

UF, Ultrafiltration; NF, Nanofiltration; RO, Reverse osmosis.

## Discussion

Using filtration membranes with a varied molecular weight cut-off (MWCO), UF, NF, and RO systems remove impurities in water. Specifically, various ions are retained in the UF-process water, and NF retains particles smaller than about 1–10 nm. A RO system almost rejects all water contaminants and ions. Tang et al. (24) analyzed drinking water softening technologies. They suggested that a NF system reduces 43–99% of calcium and 48–99% of magnesium in water, while an RO system almost removes all calcium and magnesium. Our data consistently similar calcium and magnesium contents in tap water and UF-process direct-drinking water. Their contents were significantly reduced in water samples supplied through a NF or RO system, especially in the latter. Regulations have been proposed for calcium and magnesium in drinking water in the European Union member states (25). Currently, a domestic guideline for minimum detection limits of calcium and magnesium in drinking water, especially in direct-drinking water is scant. According to the World Health Organization (WHO) standards, the minimum allowed limits of calcium and magnesium in drinking water are 20 mg/L and 10 mg/L, respectively (14). Calcium and magnesium contents in public drinking water samples collected from 314 cities in China range 2.5–155.1 mg/L and 0.2–81.9 mg/L, respectively (26). In the present study, we collected water samples from 41 primary and secondary schools in Taicang City. It is found that the calcium content in tap water and UF-process water in school exceeded the minimum allowed limit recommended by WHO, and the magnesium content was half of the recommendation. Moreover, calcium and magnesium contents in water samples supplied through a NF or RO system were lower than the recommended ranges.

Cormick et al. (27) calculated that the mean calcium availability in drinking water per person per day is 49 mg in 62 low-and middle-income countries. Our data revealed that 26.31 mg/day of calcium and 4.32 mg/day of magnesium were available in UF-process direct-drinking water in primary and secondary schools in Taicang City. In a cohort of French adults, the contribution rates of mineral water to the DI of calcium and magnesium are 25 and 6–17%, respectively (28). Drinking mineral water provides 20% of the daily calcium and magnesium intake (29). However, previous studies mainly analyzed the contribution of calcium and magnesium-rich drinking water. The present survey in primary and secondary schools of Taicang City showed that calcium and magnesium contents in tap water and UF-process direct-drinking water were relatively low. The contribution rates of direct-drinking water supplied through a UF system to the DI and RNI of calcium were 8.95 and 2.63%, respectively, and those to the DI and RNI of magnesium were 2.78 and 1.96%, respectively. The contribution of direct-drinking water supplied through a NF/RO system to calcium and magnesium intake was negligible. Similarly, drinking water offers a 1.3–1.9% of contribution to the RNI of calcium in a South American country (30). Taking into account of drinking UF-process direct-drinking water throughout the day, daily drinking water is estimated to contribute 10% of dietary calcium intake and 3% of dietary magnesium intake.

Through surveying the height data of first-grade children in Taicang City from 2019 to 2023, we did not find a significant difference in the height between UF group and NF/RO group, except for a significant difference in the height of girls between groups in 2019. The mean height growth in the cohort during 2019–2023 was slightly higher in UF group than that of NF/RO group, although a significant difference was not detectable. A cross-sectional study in an island in Zhejiang Province, China showed that there is no significant difference in the height of children and adolescents either drinking desalinated water or not (31). Meanwhile, the height of children and adolescents drinking desalinated water in this region is similar to the global level. A meta-analysis demonstrated that calcium fortified foods promote height growth and bone health in children, although they produce very little effect (32). On the contrary, an eco-epidemiological study suggested significantly greater height and height growth in children drinking mineral water in school of Chongqing city, China than those drinking low mineral water (14). We did not identify significant differences in the height between UF group and NF/RO group, which may be attributed to the low calcium and magnesium contents in UF-process direct-drinking water and the insufficient follow-up time.

Calcium and magnesium are abundant cations present in water that are easily absorbed by the human gastrointestinal tract, showing an excellent bioavailability (33). Drinking mineral water promotes bone metabolism and inhibits bone resorption via reducing the secretion of parathyroid hormone (34, 35). A retrospective cohort study illustrated an association between drinking very low mineral water and low bone mineral in children (36). Calcium supplements are recommended to promote bone mineral density (BMD) (37, 38). A systematic review unveiled that the calcium intake in Chinese children and adolescents at 0–17 years of age ranges 236–801 mg/day, with the prevalence of inadequate intake inflated to 92–96%. Meanwhile, the daily magnesium intake in Chinese children and adolescents at 0.5–17 years of age is 112–369 mg, presenting a 50–60% of inadequate intake rate (39). Calcium and magnesium intake of Taicang residents are 250 mg/day and 240 mg/day, respectively, and the intake of children and adolescents is slightly higher (20). Calcium intake below 300 mg/day during adolescence may result in a shorter adult stature (40). The dietary intake of calcium and magnesium is generally insufficient in children and adolescents in Taicang City, and a further reduced intake of calcium and magnesium in drinking water may retard height growth. Kozisek (41) and Verma et al. (42) proposed that the use of desalinated soft water for cooking leads to a massive loss of calcium and magnesium in food ingredients, further reducing dietary intake. Consumption of very low-mineral water may retard height (14) and threaten cardiovascular health (43) in children. A study showed that milk intake can promote children’s growth more than calcium supplementation (44). A randomized controlled trial showed that calcium supplementation of boys in late childhood advances the age of peak height velocity and results in a shorter adult stature in children on habitually low calcium intakes (22). Recently, a rising body of evidence raises concerns about calcium supplements due to their potential risks to cardiovascular health (45, 46). Therefore, we believed that obtaining minerals from natural foods or water rather than supplements is the optimal approach. For school-age children, obtaining minerals like calcium and magnesium through drinking water is a safe, economic and convenient way.

Our study had certain strengths. To our knowledge, this is one of the few studies on the contribution of direct-drinking water to calcium and magnesium and the influence on the height in school-age children. We conducted a comprehensive analysis from the calcium and magnesium contents of direct-drinking water, to the contribution rates, and finally to the influence of children’s height. In addition, we included all eligible first-grade children in the retrospective cohort, who could be representative of the local population. Although we did not obtain significant differences in the height and height growth between UF group and NF/RO group, calcium and magnesium supplements from direct-drinking water in school may provide benefits to school-age children.

Limitations in the present study should be noted. First, the calculation of contribution rates relies on the *Nutrition and Health Status Survey 2021* rather than the retrospective cohort, which may not reflect the real situation of children in the cohort. Second, we failed to control confounding factors (e.g., mid-parental height, socioeconomic status, mineral supplements, and physical activities) and analysis of sensitive biological indicators of the height (e.g., blood calcium, blood magnesium, parathyroid hormone, and BMD). Dietary and water mineral intakes of children in the cohort are another important confounding factors that we did not obtain. Considering the children included in this study lived in the same city and shared similar dietary habits, they may have similar intakes of dietary minerals. Besides, a total of 740 children missed physical examination or followed-up, accounting for 13.2% of the initial participants, which may lead to overestimation or underestimation of results. We believed that more large-scale studies involving children and adolescents with a follow-up until adulthood are needed. Confounding factors should be strictly controlled and more sensitive biomarkers should be detected to make the results significant and robust.

## Conclusion

The calcium and magnesium contents in tap water are similar to those in UF-process direct-drinking water, but significantly reduce in water samples supplied through a NF or RO system. The contribution of direct-drinking water supplied through a UF system to calcium and magnesium intake is not negligible. The mean height growth in the cohort of primary school children during 2019–2023 is slightly higher in UF group than that of NF/RO group, but a significant difference is not detectable. Filtration of harmful components while retaining beneficial components in direct-drinking water and its impact on health are the future spotlight of research. Direct-drinking water processed through a NF or RO system should be cautiously adopted in primary and secondary schools. A UF system is preferred in schools where no health concerns are associated with water quality.

## Data Availability

The original contributions presented in the study are included in the article/supplementary material, further inquiries can be directed to the corresponding authors.
